# Next in Surgical Data Science: Autonomous Non-Technical Skill Assessment in Minimally Invasive Surgery Training

**DOI:** 10.3390/jcm11247533

**Published:** 2022-12-19

**Authors:** Renáta Nagyné Elek, Tamás Haidegger

**Affiliations:** 1 Antal Bejczy Center for Intelligent Robotics, University Research and Innovation Center, Óbuda University, H-1034 Budapest, Hungary; 2 Doctoral School of Applied Informatics and Applied Mathematics, Óbuda University, H-1034 Budapest, Hungary; 3 John von Neumann Faculty of Informatics, Óbuda University, H-1034 Budapest, Hungary; 4 Austrian Center for Medical Innovation and Technology, 2700 Wiener Neustadt, Austria

**Keywords:** minimally invasive surgery, robot-assisted minimally invasive surgery, surgical skill assessment, non-technical skills, surgical data science, workload, situation awareness, autonomous skill assessment

## Abstract

Background: It is well understood that surgical skills largely define patient outcomes both in Minimally Invasive Surgery (MIS) and Robot-Assisted MIS (RAMIS). Non-technical surgical skills, including stress and distraction resilience, decision-making and situation awareness also contribute significantly. Autonomous, technologically supported objective skill assessment can be efficient tools to improve patient outcomes without the need to involve expert surgeon reviewers. However, autonomous non-technical skill assessments are unstandardized and open for more research. Recently, Surgical Data Science (SDS) has become able to improve the quality of interventional healthcare with big data and data processing techniques (capture, organization, analysis and modeling of data). SDS techniques can also help to achieve autonomous non-technical surgical skill assessments. Methods: An MIS training experiment is introduced to autonomously assess non-technical skills and to analyse the workload based on sensory data (video image and force) and a self-rating questionnaire (SURG-TLX). A sensorized surgical skill training phantom and adjacent training workflow were designed to simulate a complicated Laparoscopic Cholecystectomy task; the dissection of the cholecyst’s peritonial layer and the safe clip application on the cystic artery in an uncomfortable environment. A total of 20 training sessions were recorded from 7 subjects (3 non-medicals, 2 residents, 1 expert surgeon and 1 expert MIS surgeon). Workload and learning curves were studied via SURG-TLX. For autonomous non-technical skill assessment, video image data with tracked instruments based on Channel and Spatial Reliability Tracker (CSRT) and force data were utilized. An autonomous time series classification was achieved by a Fully Convolutional Neural Network (FCN), where the class labels were provided by SURG-TLX. Results: With unpaired *t*-tests, significant differences were found between the two groups (medical professionals and control) in certain workload components (mental demands, physical demands, and situational stress, p<0.0001, 95% confidence interval, p<0.05 for task complexity). With paired *t*-tests, the learning curves of the trials were also studied; the task complexity resulted in a significant difference between the first and the second trials. Autonomous non-technical skill classification was based on the FCN by applying the tool trajectories and force data as input. This resulted in a high accuracy (85%) on temporal demands classification based on the *z* component of the used forces and 75% accuracy for classifying mental demands/situational stress with the *x* component of the used forces validated with Leave One Out Cross-Validation. Conclusions: Non-technical skills and workload components can be classified autonomously based on measured training data. SDS can be effective via automated non-technical skill assessment.

## 1. Introduction

The conversion to Minimally Invasive Surgery (MIS) from open access surgery was one of the most important paradigm changes in medicine in the second half of the 20th century [1]. Despite the clear benefits of MIS, such as shorter recovery times, smaller scars, and less blood loss, it can also pose a challenge to surgeons by the limited view, the loss of depth information due to the 2D endoscopic camera, but especially by the complicated handling of the laparoscopic tools. MIS requires extensive training; and because of the challenging tasks, constant feedback regarding skills is needed as well [2].

**Figure 1:**
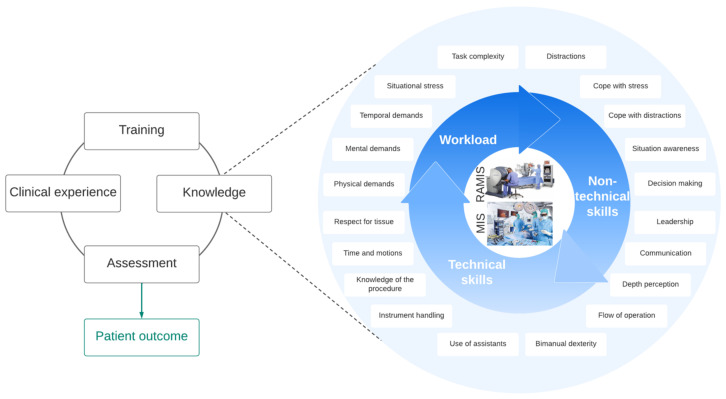
Surgical skills in MIS and RAMIS are built on technical skills, non-technical skills and managing the workload. These segments are directly influencing each other. Surgical skill training and assessment can affect the skills of the surgeon, which derive patient outcomes.

Surgical skills and knowledge directly influence patient outcome, which are based on years of training, supervisory assessment and clinical experience. The knowledge of the surgeon includes technical skills, non-technical skills, and workload (Figure 1). Assessing the objectively measured technical skills of the surgeon (such as tool handling, bimanual dexterity, the flow of operation, etc.) is a very well-researched domain; non-technical skills (situation awareness, dealing with stress and distractions, decision-making, etc.) can be similarly important considering the patient outcomes [3]. Workload, which represents the effort to perform a task, may also be in strong correlation with non-technical skills. In many parts of the world, surgical skill training and assessment are not part of the clinical practices.

Surgical skills can be assessed with self-rating questionnaires, where the performance is self-evaluated by the surgeon, and standardized expert rating techniques, where an expert panel (typically 8–10 surgeons) evaluates the surgical procedure (or surgical training) based on a video recording. Both of these approaches can inherently be biased, and they require significant human resources [4]. The general aim is to automate surgical skill assessments since they are objective, but technical backgrounds and the key surgical features and factors are still under intensive research. Surgical Data Science (SDS) aims to improve the quality of interventional healthcare with data science techniques, including capture, organization, analysis, and modeling of data. With SDS techniques, automated surgical skill assessment can be achieved, and the key skills can be verified with the correlations between them [5].

Robot-Assisted Minimally Invasive Surgery (RAMIS) denotes the surgical technique, where the robot-applied laparoscopic tools are remotely controlled by a human operator at a console. Teleoperation can help surgeons with tremor filtering, 3D endoscopic vision, ergonomy, re-scaled motion, and easier tool handling. RAMIS skill assessment is a well-studied area, because in this case, sensory data are readily available (robot kinematic data and 3D video endoscope). RAMIS skill assessment is closely related to MIS, the key factors are very similar in technical and non-technical skills as well [2,4].

## 2. Aims and Scope

This paper reports on the first part of a two-stage research, where autonomous MIS non-technical skill assessment is examined. The goal of our work was to improve non-technical skill training and examine correlations between objectively measured parameters and workload, which can improve the surgical outcome. In this paper, an automated, sensory data-based (video image and force input) non-technical surgical skill assessment method is introduced. The idea behind the study was to utilize SDS techniques for autonomous non-technical skill assessment. We aimed to understand the correlations between tool motions, used forces, and situation awareness, in the case of a stressful clinical situation with distractions.

- For the experiments, a surgical training environment and workflow were designed where non-technical skill assessment is addressed; a sensorized MIS training platform was designed and created, which simulates specific parts of Laparoscopic Cholecystectomy: (1) safe clipping of the cystic artery and (2) the dissection of the parietal peritoneal layer;
- A workload analysis with SURG-TLX was performed with different subjects to understand the key workload factors and statistical differences between the groups;
- An autonomous non-technical skill assessment method was developed based on sensory data, where the class labels were the results from SURG-TLX. With this, correlations between sensory data and non-technical skills can be better understood.

## 3. Related Work

In the literature, workload assessment is typically based on self-rating questionnaires. While self-rating is a straightforward way to assess workload, it can be biased. An often-used questionnaire, the NASA-TLX, has already been employed for workload assessment to understand the physical and mental efforts in MIS [6,7] and in RAMIS [8,9] as well. NASA-TLX assesses the workload based on mental, physical, and temporal demand, effort, performance, and frustration level. A surgery-related modified version of NASA-TLX is SURG-TLX, which considers distractions and stress. SURG-TLX for workload assessment in MIS can be found in the literature [10,11], but in the case of RAMIS, it is still not a well-established approach, only a few studies utilized this method [12,13,14].

Expert rating is a frequently used technique for surgical skill assessment because of its objectivity (compared to self-rating). However, it requires an expert surgeon panel, who can benchmark the performance based on a validated technique. While this process can still show some bias, in most cases, this approach is called "objective" in the literature. On the other hand, expert rating can be a very good solution identifying class labels and scores for autonomous assessment. Validated expert rating techniques can already be found in the literature: Non-Technical Skills Scoring System (NOTECHS) mainly considers teamwork via team skills, communication, interaction, and management skills, but it also assesses decision-making and situation awareness [15]. Observational Teamwork Assessment for Surgery (OTAS) assesses mainly teamwork too [16]. Non-Technical Skills for Surgeons (NOTSS) considers decision-making, situation awareness and stress besides teamwork [17]. The only expert rating non-technical skill assessment tool for RAMIS is the Interpersonal and Cognitive Assessment for Robotic Surgery (ICARS) [18]. While MIS expert rating systems are applied in many studies, ICARS has not been recognized widely to date.

Autonomous skill assessment is a well-studied domain with respect to technical skills; it is the most objective, and does not require human resources; however, its technical implementation is more difficult. In technical skill assessment, there are two main approaches: kinematic data-based [19] and image-based [20] methods. Autonomous non-technical skill assessment is less standardized compared to technical skill assessment. The objective ways to generating data, which can be autonomously scored or classified to measure non-technical skills, are still being researched. In the meanwhile, SDS is focusing on understanding the key parameters of surgery based on data; thus it can provide a possible solution for non-technical skill assessment in an autonomous way. Data generation for skill classification can be conducted by physiological signals, such as brain activity [21,22], skin temperature [23,24], eye movement [25,26], blood pressure [27], etc., which are related to stress. These represent only one dimension of non-technical skills, and still can be influenced by other factors, such as personal health. Electroencephalogram (EEG) [8], Functional Near-Infrared Spectroscopy (fNIRS) [14,28] and heart rate (HR) [29] are the most studied sensory inputs for non-technical skill assessment. In the case of RAMIS, built-in sensor usage is also a possibility for non-technical skill assessment. These can provide kinematic and video data, such as with encoders and endoscopic cameras. The spread of the RAMIS systems can open new possibilities in SDS and autonomous non-technical surgical skill assessment, especially with more than 7500 da Vinci Surgical Systems (Intuitive Surgical Inc., Sunnyvale, CA) worldwide, and the da Vinci Research Kit (DVRK) [30] community, which is focusing on RAMIS research [31].

## 4. Methods

The chosen MIS training task requires well-maintained situation awareness, dealing with stress and distractions, and prompt decision-making. A total of 20 experiments were conducted by 7 subjects including residents with different expertise levels, expert surgeons, and a control group. The training task was to perform certain steps of laparoscopic cholecystectomy, as the dissection of the peritoneal layer without damaging the cholecyst, and during the procedure, abrupt bleeding was induced as a complication, which had to be handled by the subjects. The workload analysis was done with a self-rating questionnaire (Surgery Task Load Index, SURG-TLX) [32], which is a modified version of the well-known NASA-TLX, which was originally created for assessing the workload of pilots. NASA-TLX is an effective tool in other research domains, such as in clinical medicine [33]. Statistical differences between the workload were examined between the groups, and the learning curve was also assessed. For autonomous non-technical skill assessment, video image and force data were used as input. Tool motion was processed with an object tracking computer vision algorithm (Channel and Spatial Reliability Tracker (CSRT) [34]). The time series data (force and image) were classified with a Fully Convolutional Neural Network-based classifier [35], where the class labels were the results from SURG-TLX.

### 4.1. Medical Background

Laparoscopic Cholecystectomy (LC) is one of the most common interventions with more than 300,000 operations performed in the US annually [36]. The main indications are gallstones (cholecystolithiasis), inflammation of the gallbladder (cholecystitis), or less frequently, polyp or neoplasm of the gallbladder itself. Cholecystectomy is mainly done by the gold-standard laparoscopic method, especially in elective cases. Nevertheless, LC still requires significant training since damage in the hepato-biliary tract can cause serious complications.

**Figure 2:**
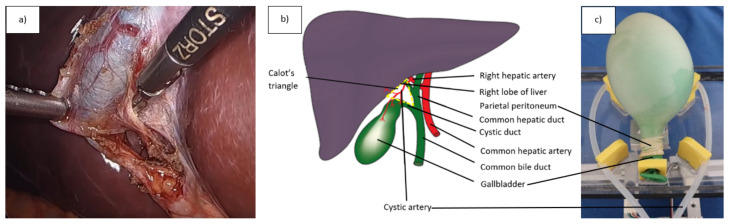
Laparoscopic Cholecystectomy (LC) anatomy and the proposed phantom; (**a**) Laparoscopic Cholecystectomy surgical scene, after the exploration of the Calot’s triangle, which is a critical task in patient safety; (**b**) Anatomy of the gallbladder and its environment; (**c**) Surgical phantom created for LC with the peritoneum, gallbladder and the cystic artery, which provides the option for abrupt bleeding, intending to mimic a stressful surgical situation.

LC’s one important phase, which requires good non-technical skills, is exposing Calot’s Triangle’s structures at the porta hepatis (Figure 2), including the following steps:

- Localization;
- Dissection;
- Clipping;
- Transecting the cystic artery and cystic bile duct (maintaining the attention is critical to avoid the choledochal duct and duodenum itself).

Failure in any of these steps would cause intra-operative or post-operative severe bleeding, injury or damage of nearby organs or bile leakage with the necessity of re-operation and High Dependency Intensive Care, caused by general inflammation of the peritoneum and systematic septic condition. LC involves the dissection and cauterisation of the parietal peritoneal layer to remove the gallbladder from its “nest” attached to the liver itself.

### 4.2. Surgical Phantom

Presenting a training environment and a surgical phantom, where non-technical skills can be examined was the first goal of the research. Our aim was to create a phantom which represents the gallbladder/cholecyst and its environment for LC. Besides anatomical similarity and port-entry arrangement, laparoscopic tool motions should be similar to a real surgery during the training; as this study aimed to assess non-technical skills, stressful situations as adverse events had to be involved via the phantom. To achieve this, a simple surgical training phantom for LC was created based on a gallbladder dissection phantom (Laparoscopyboxx, Nijmegen, NL) [37]. In this phantom (Figure 2), a balloon (green) was placed inside another balloon (translucent), which was then blown. The outer balloon (representing the peritoneal layer) should be dissected without causing damage to the inner balloon (representing the cholecyst). A pre-damaged, droplet leaking tube was added to the model, which represented the cystic artery clipping failure aiming to create a stressful situation which required quick decision-making.

### 4.3. Workflow of the Experiment

**Figure 3:**
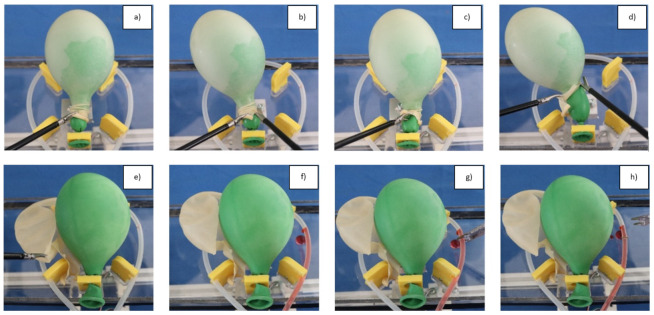
Proposed workflow of the MIS experiment; (**a**) Grasping the outer layer (representing the parietal peritoneum) with a dissector; (**b**) Cutting the outer layer with a pair of scissors; (**c**) Blunt dissection; (**d**) Cutting; (**e**) Removing the covering layer; (**f**) Abrupt bleeding; (**g**) Localize the bleeding source, change the tool(s) to the clipper; (**h**) Clipping the blood vessel considering the direction of the blood flow.

Three laparoscopic tools were used in the study: a dissector, a pair of scissors, and a 10 mm single-use clipper. All experiments started with a short theoretical introduction of the surgical domain to the subjects and a video illustration of the task. For the control group, the proper usage of the MIS tools was also shown. To simulate an MIS environment, it was essential to implement the experiments in a laparoscopic box trainer, where two entry ports were used for the two training tasks: dissecting the outer balloon with the dissector and the scissors, and clipping the bleeding blood vessel. The operating area was visualized by a camera, where the two-dimensional RGB images were shown to the participants on a screen. Because the aim was to create limited ergonomy, the screen was placed beside the trainer box, where the subjects could only see the camera image in an uncomfortable posture. The subjects could perform blunt dissection, cutting, and grasping with the given tools during the dissection task (Figure 3), but the exact technique was not defined. The workflow of the experiments were built from the following tasks:

- Dissection of the outer layer without damaging the inner object (which determines if the procedure was successful or not);
- Localization of the abrupt bleeding;
- Remove one tool (or both), and change then to a clip applier;
- Clipping the artery, considering the direction of the blood flow.

The trials were rushed after the bleeding occurred to create a stressful environment. During the whole procedure, disturbing factors (speaking around the operator, noises) were introduced simulating a real-time operating room. The subjects had to fulfill all of the tasks three times; after each task, they had to fill out a questionnaire (SURG-TLX) about the experienced workload. Following the experiments, medical professionals also filled out a questionnaire about the reality of the used phantom and the surgical environment. Their simulator and video game usage were also surveyed.

### 4.4. Examining Workload: SURG-TLX

Every participant performed the phantom-LC surgery three times, except one subject. There were 20 training procedures recorded from 7 subjects during the experiment. After every session, the subjects had to fill out a questionnaire about the experienced workload. As it was introduced, SURG-TLX (created the cooperation between the University of Hong Kong, the University of Exeter and the Department of Urology, Royal Devon, and Exeter Hospital in 2011) is a modified NASA-TLX metric for surgical workload assessment [32]. SURG-TLX (Table 1) assesses the workload by considering:

- Mental demands;
- Physical demands;
- Temporal demands;
- Task complexity;
- Situational stress;
- Distractions.

At the end of the experiment, medical professionals scored the reality of the surgical phantom and the experiment based on the experiment’s applicability for MIS training, the surgical instrument movements similarity to real laparoscopic surgery, the anatomical similarity and the experiment’s capability to simulate stressful surgical situations (Table 2).

### 4.5. Hardware and Software Environment

**Figure 4:**
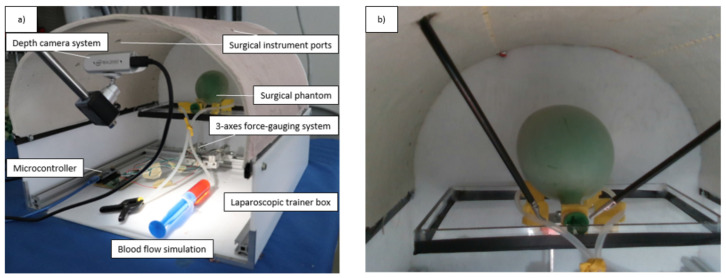
Experimental environment; (**a**) MIS box trainer with the components of the experiment–phantom and sensors, such as the camera and the force-gauging system); (**b**) Camera image streamed for the operators during the training and recorded for data processing.

To provide sensory data, camera images and force sensor data were used. For the camera stream, Intel Realsense D435i’s RGB channel images were saved (Figure 4). An additional camera was used, since the DVRK Endoscope Camera Manipulator (ECM) is attached to a da Vinci Classic, which is a first-generation RAMIS system, and its endoscopic camera provides inferior image quality. Because of this, no real endoscopic camera was used in this study (except its lighting). Since this work does not focus on implementing new image processing algorithms for the da Vinci Classic, the usage of an additional camera can provide good-quality images without dealing with the endoscopic camera stream. Furthermore, in most of the cases, the accuracy of the depth data is better with active stereo compared to passive stereo systems, which can be an input in future studies. The reason for only using the 2D image stream was to simulate the manual and mostly widespread 2D capable MIS laparoscopic systems, where it is only available; but since in future research, RAMIS is considered, 3D stream was also recorded. The forces exerted during the training were measured with a force-gauging system that measured the resultant external forces acting on the surgical phantom along three perpendicular axes. The main program was written in Python 3 programming language, with OpenCV 4, Keras, and TensorFlow 2 libraries utilized for computer vision and machine learning. The main program was responsible for the following tasks:

- RGB images streaming;
- Force and camera image data synchronization;
- Calculation of the two-dimensional position of the surgical instruments;
- Save the time series data (synchronized force and image) into a Deep Neural Network-readable format, and performing the classification.

### 4.6. Subjects

In this study, we report 20 LC training conducted by 7 subjects in the proposed experimental environment: 3 non-medical subjects as a control group, 1 very experienced resident surgeon with 50+ h in MIS, 1 experienced surgery resident with 31–50 h in MIS, and two expert surgeons. All participants have done the training several times (3 for each, except 2 for one of the expert surgeons). Both male and female subjects were included in the groups, each subject was right-handed. The subjects were between 25–61 years with mean age 46 years. The residents had experience in MIS training in a laparoscopic box trainer (with 2 to 50 h). Participants had very little or no experience in video gaming. None of the participants had experience in robotic surgery. Heterogeneous data were required to represent the three main skill classes of surgeons (novice, intermediate, and expert).

### 4.7. Surgical Instrument Tracking

The 2D RGB images provided the input for surgical instrument tracking, which was then utilized for creating a 2D tool trajectory for autonomous skill classification. The tracking was done autonomously with Channel and Spatial Reliability Tracker (CSRT) [34]. While the tracking was autonomous for the first frames, the Region of Interest (RoI) had to be selected manually. In CSRT, an automatically estimated spatial reliability map restricted the discriminative correlation filter to the parts of an object suitable for tracking with improving the search range and performance for irregularly shaped objects. Channel reliability weights calculated in the constrained optimization step of the correlation filter learning reduced the noise of the weight-averaged filter response [34]. CSRT is a very powerful tool for RoI tracking, it worked robustly in the case of the training videos, but in the case of large-scale motions, the implemented method made RoI re-selection possible.

### 4.8. Autonomous Skill Classification

For every trial (20), time series were built based on image and force data, containing the following 9 parameters:

- Dissector *x* and *y* coordinates (dissector${}_{xy}$);
- Scissors *x* and *y* coordinates (scissors${}_{xy}$);
- Clipper *x* and *y* coordinates (clipper${}_{xy}$);
- Synchronized force data in *x*, *y* and *z* directions (force${}_{xyz}$).

**Figure 5:**
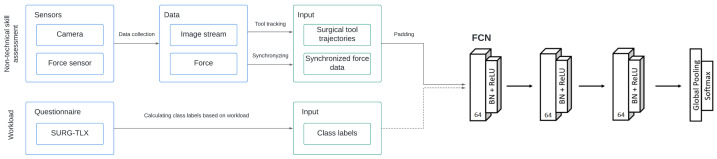
Autonomous non-technical surgical skill assessment method workflow based on sensory data with FCN multivariate time series classification.

The longest trial was 94,068 frames long, which was the template length for padding the other trials time series to provide a time series classifier-readable format (same length for every trial). For autonomous skill classification based on multivariate time series, a Fully Convolutional Neural Network (FCN) was utilized. The used FCN architecture was built from a convolutional layer, followed by a batch normalization layer and a Rectified Linear Unit (*ReLU*) activation layer [35] (Figure 5). The convolution operation was conducted by three 1D kernels. The convolution block was built from a convolutional layer:

(1) y=W⊗x+b,

where *y* denotes the output of the convolutional layer, calculated from the weights of the neurons (*W*) and bias (*b*) with a convolution operation (⊗). Y is the input of the batch normalization layer (*BN*):

(2) s=BN(y),

and based on the output of it (*s*), the output of the ReLU activation layer (*h*) can be calculated:

(3) h=ReLU(s).

The network was built by stacking three convolution blocks with filter sizes (64, 64, 64) in each block. The features were then fed into a global average pooling layer, and the final label was produced by a softmax layer. The output of the network was a binary class label.

## 5. Results

### 5.1. Outcome Results

The first task—which was the dissection of the parietal peritoneum layer—was denoted successful when the gallbladder was not harmed. Of the 20 trials, 7 were successful. One novice from the control group successfully dissected the outer layer twice; the very experienced resident performed the task successfully for the second time; the expert surgeon was able to conduct the dissection task the second time, and the expert MIS surgeon performed the task successfully for all three cases. The second task—which was abrupt bleeding handling with changing at least one tool to the clipper, and clipping the vein considering the direction of the blood flow—was conducted successfully by every subject.

### 5.2. Workload Results

**Figure 6:**
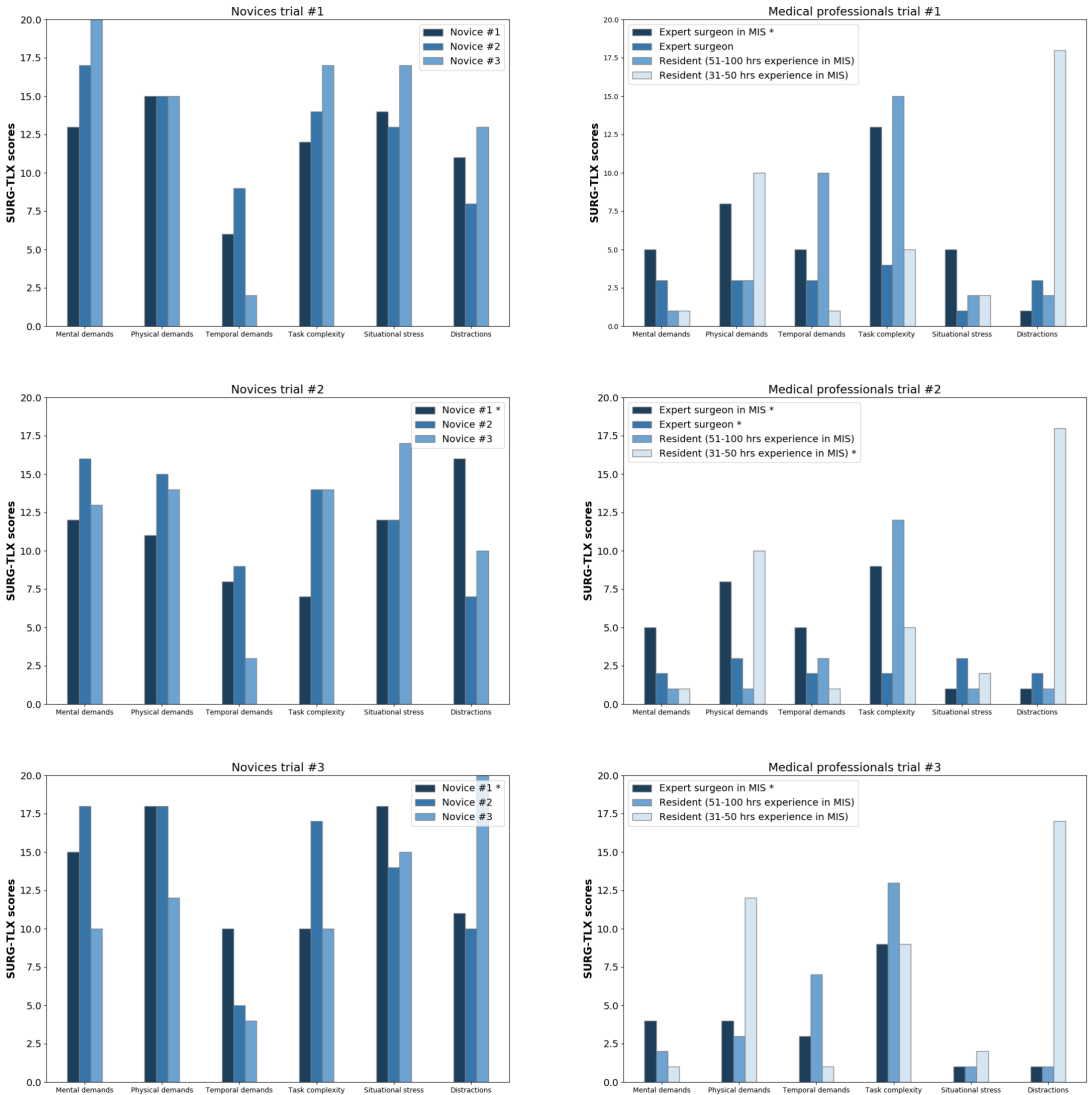
SURG-TLX values (0–20) of medical professionals from first to third trials (first column) and novices (second column). Asterisk (*) notes if the first task (dissecting the cholecyst) was successful.

Workload scores were provided by SURG-TLX. Unpaired *t*-tests were used to determine statistical differences between medical professionals and the control group in the case of workload (Table 3, Figure 6). From SURG-TLX, in the case of mental demands, physical demands and situational stress high significance was found between the medical professionals and the control group (p<0.0001, 95% confidence interval), a significant difference (p<0.05) in the case of task complexity, and no significant differences in the case of temporal demands and distractions. The workload learning curve was tested with paired *t*-tests between trials 1–3 (Table 4). No significant differences were found, except for task complexity, which showed significant deviations in the workloads between the first and the second trials (p<0.05). Because all trials (3) used for autonomous skill assessment studies, it was necessary to understand if there were significant differences between the trials. Furthermore, the differences between task complexity suggest that the proposed environment can train that aspect of workload; however, we can not conclude it from this limited amount of data.

### 5.3. Phantom Validation

**Figure 7:**
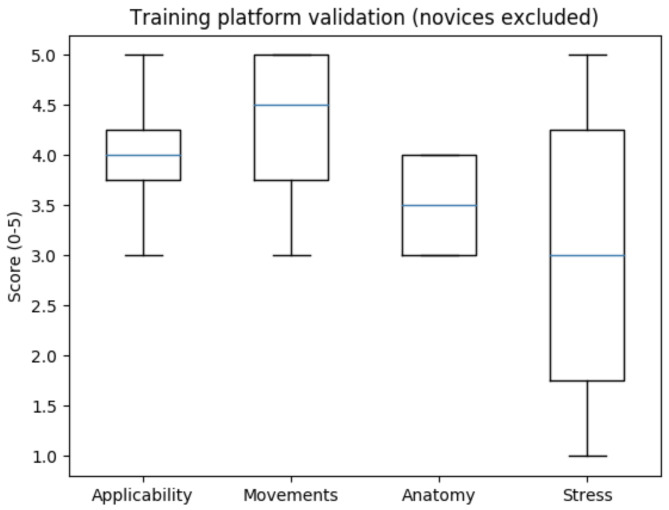
Surgical training environment and phantom validation (applicability for laparoscopy training, similarity of motions, anatomical similarity, simulation of stress) based only on medical professionals’ evaluation. The environment was assessed as good for applicability, very good for movement similarity, acceptable for anatomical similarity and moderate for stress simulation.

Since the phantom and the training environment were custom-designed, they had to be validated by medical professionals. It was completed based on a questionnaire, including the questions introduced in Table 2. The environment was assessed as good for applicability (mean value: 4/5), very good for movement similarity (mean value: 4.5/5), acceptable for anatomical similarity (mean value: 3.5/5), and moderate for stress simulation (mean value: 3/5) (Figure 7).

### 5.4. Autonomous Non-Technical Skill Assessment based on Time Series Data

The 9-parameter time series data (Section 4.8) were binary classified by an FCN. For training and testing, the binary class labels were defined by SURG-TLX; scores below average were defined as *class 0*, and results above the average were indicated as *class 1* label. With this technique, the class labels were calculated in the same way for experience (medical professionals/control group), mental demands and situational stress. In the meanwhile, the class labels were calculated differently for physical demands, temporal demands, task complexity, distractions and outcome (which defined whether the dissection was successful or not). The usage of class labels from SURG-TLX allows studying of workload components, which are not necessarily the same as technical experience. By this, the correlation between the workload and the objectively measured data can be better examined.

The classification was tested independently for the force data along *x*, *y* and *z* axes (force${}_{x}$, force${}_{y}$, force${}_{z}$) and for the laparoscopic instruments’ position in the image space (dissector${}_{xy}$, scissors${}_{xy}$, clipper${}_{xy}$). and the combinations of the parameters (force data and all instruments’ path, dissector with scissors path and force data, dissector with scissors path with force${}_{z}$) were tested as well.

Autonomous skill classification results were validated with Leave One Out Cross-Validation (LOOCV). In the case of LOOCV, each observation was considered as the validation set and the rest of the observations were considered as the training set; this was iterated through the whole dataset, which means every observation was considered independently. Next, the results were averaged for the 20 cases. The accuracy averages can be found in Table 5 and Table 6. The best result was provided for temporal demands based on the *z* component of the used forces (85% accuracy). The classification of temporal demands showed higher overall accuracy compared to other metrics based on the path of the dissector and the scissors with the *z* component of the used forces (70% accuracy) or other combinations. Higher accuracy (75%) result was found in the case of classifying mental demands/situational stress with the *x* component of the used forces. For the other cases, moderate or low accuracy was found in the classification.

## 6. Discussion and Future Work

Our future research will focus on RAMIS non-technical skill assessment under the same conditions (research protocol, surgical phantom, workflow, and methodology). Since autonomous non-technical skill assessment is not a very well-studied research domain in RAMIS [3], we plan to compare and validate results in RAMIS with results in MIS.

Self-rating techniques can be powerful tools for workload analysis and to provide class labels for non-technical skill assessment. Since they do not require human resources, they can be subjective. In some cases, self-rating rather correlates with confidence instead of real skills. Self-rating is a very easy way to create class labels for skill classifications, to test the autonomous solutions, understand the key factors, demands, and stressors in surgery, and validate a surgical training environment. This work presented an autonomous non-technical skill assessment method with FCN based on sensory data. Tool motions and applied forces were examined in technical skill assessment most often, yet, our aim was to prove that these sensory data are also linked to non-technical skills. For this, class labels from SURG-TLX were used, where all workload components (physical demands, temporal demands, task complexity, etc.) were studied, which were independent from the technical experience (except in the case of mental demands and situational stress, where the class labels based on SURG-TLX resulted in the same as technical knowledge). With this method, the correlation between the workload and the objectively measured data could be examined, nevertheless, the class labels were created by a self-rating questionnaire. In this study, for tool tracking, 2D image data were used with an object tracking computer vision algorithm. While it worked robustly, in the case of image coordinates usage, there is a dimension loss, since these positions are available only in the image space. 3D position and 3D orientation of the surgical instruments can provide higher accuracy for skill classification, furthermore, other kinematic data (acceleration, velocity, etc.) can also improve the precision of the assessment. In the case of traditional MIS, it is achievable with additional sensors or with an advanced image processing solution. In the case of RAMIS, kinematic data for all robotic arms may be available, e.g., with the usage of the DVRK. Furthermore, in RAMIS, there is a 3D video stream provided by the stereo endoscope, where stereo image processing, such as surgical instrument segmentation can be employed for more data.

For basic statistical analysis, the amount of data collected was sufficient, however, for a neural network-based classification, the more data, the better to achieve a general convergence; thus, the number of subjects should be increased in the future. In the case of the phantom validation tests, especially the question about the phantom representation of stress, the answers showed a large standard deviation, which could also be more obvious with more involved subjects. In the future, we intend to repeat this study with more participants; furthermore, the same experiment will be done with the da Vinci Surgical System. By this, the workload and non-technical skills could be compared in the case of MIS and RAMIS in stressful surgical training tasks. Since RAMIS provides 6+1 Degrees of Freedom for the surgical instrument, ergonomy, and 3D vision, less workload and better skills are expected. In the meanwhile, handling a robotic platform that requires training may cause stress for the operator. Furthermore, changing the tools in the case of RAMIS is not done by the operator, it is the work of an assistant; thus, changing clippers requires teamwork and communication skills from the operator, as well as good non-technical skills (quick decision-making) from the assistant as well.

There are studies in the literature (discussed in the Introduction Section) that use objectively measured data for workload and non-technical skill assessment. Unfortunately, these studies are very heterogeneous; therefore, the results of other studies cannot be compared here. Nevertheless, in our future work, a MIS and RAMIS surgical dataset will be created and annotated for non-technical skill assessment. JHU–ISI Gesture and Skill Assessment Working Set (JIGSAWS) is a good example of this, which is a RAMIS dataset annotated with technical skills [38]. By this, the results will be made comparable.

## 7. Conclusions

In this paper, autonomous non-technical skill assessment and workload analysis were presented for MIS training. The chosen surgical procedure was Laparoscopic Cholecystectomy, especially focusing on the dissection of the parietal peritoneum and clipping the cystic artery. Stressful surgical situations were generated by a long, tiring, and possibly dangerous dissection, abrupt bleeding, which required quick solution, and a rushed training with disturbing factors. A surgical training platform and workflow were designed, which included the cholecyst, the parietal peritoneum layer, and the cystic artery. The surgical phantom was extended with additional sensors (force and image) to acquire objectively measurable surgical parameters. 20 experiments were concluded by 7 subjects with different expertise levels from novices to expert surgeons. The workload was analysed by SURG-TLX, which provided class labels for FCN-based skill classification as well. Unpaired *t*-tests showed significant differences between the various skill levels in the cases of mental demands, physical demands, situational stress and task complexity. The learning curves in workload studied with paired *t*-tests showed significant differences in the case of task complexity between the first and the second trials. The surgical instruments were tracked on 2D images with a CSRT tracker, creating image-based time series data for all trials. Then, force data were synchronized with image data, creating a nine-parameter time series data. This dataset was used to train and test skill classification with a FCN, where the class labels were based on workload. The results were validated with LOOCV. Autonomous non-technical skill classification with FCN resulted in high accuracy on temporal demands based on the *z* component of the used forces (85 %) and 75 % accuracy for classifying mental demands/situational stress with the *x* component of the used forces. Based on these findings, with SDS techniques, certain components of non-technical skills of the surgeon can be assessed autonomously based on sensory data only. The results showed very good classification accuracy for temporal demands. It suggests how well the operator handled the rushing, the procedure can be seen in the objectively measured sensory data (especially in the case of force data). This correlates with the subjective opinion of medical professionals as well: according to a resident, they felt the experiment very rushed, and while based on the experience/mental demands/situational stress the subject was classified a 1, for temporal demands the class label was 0 based on the questionnaire. From that, it can be concluded that experience can help deal with stress, but it is not necessarily related to temporal demands, which can be autonomously measured with high accuracy.

## Figures and Tables

**Table 1:** SURG-TLX mental workload self-rating questionnaire [32].

Title	Endpoint	Description
Mental demands	low/high (0–20)	How mentally fatiguing was the procedure?
Physical demands	low/high (0–20)	How physically fatiguing was the procedure?
Temporal demands	low/high (0–20)	How hurried or rushed was the pace of the procedure?
Task complexity	low/high (0–20)	How complex was the procedure?
Situational stress	low/high (0–20)	How anxious did you feel while performing the procedure?
Distractions	low/high (0–20)	How distracting was the operating environment?

**Table 2:** Surgical phantom and training environment validation questionnaire.

Title	Endpoint	Description
Experiment’s applicability	low/high (0–5)	How appropriate is the experiment to teach MIS during the modeled surgery?
Movement similarity	low/high (0–5)	How similar are the movements to those required during surgery?
Anatomical similarity	low/high (0–5)	How realistic is the anatomical phantom designed to model the surgical area?
Simulation of stress	low/high (0–5)	How suitable is the anatomical phantom to simulate stressful surgical situations?

**Table 3:** Statistical differences between medical professionals (MP) and the control group (CG) in the case of workload, based on SURG-TLX. Mean and standard deviation (SD) values are reported for both groups. *p* values are signed as MP/CG where MP and CG groups were tested. * 0.01 < *p* < 0.05 (statistically significant); ** *p* < 0.0001 (statistically extremely significant); not signed: statistically not significant.

SURG-TLX	MP Mean	MP SD	CG Mean	CG SD	*p* Value (MP/CG)
Mental demands	2.36	1.63	14.89	3.18	0.0001 **
Physical demands	7.41	3.89	14.78	2.33	<0.0001 **
Temporal demands	3.73	2.83	6.22	2.91	>0.05
Task complexity	8.73	4.27	12.78	3.35	<0.05 *
Situational stress	1.91	1.22	14.67	2.24	<0.0001 **
Distractions	5.91	7.58	11.78	4.06	>0.05

**Table 4:** Learning curve assessment between 1st to 2nd, 2nd to 3rd and 1st to 3rd trials. * 0.01 < *p* < 0.05 (statistically significant); not signed: statistically not significant.

SURG-TLX	*p* Value (1st to 2nd)	*p* Value (2nd to 3rd)	*p* Value (1st to 3rd)
Mental demands	>0.05	>0.05	>0.05
Physical demands	>0.05	>0.05	>0.05
Temporal demands	>0.05	>0.05	>0.05
Task complexity	<0.05 *	>0.05	>0.05
Situational stress	>0.05	>0.05	>0.05
Distractions	>0.05	>0.05	>0.05

**Table 5:** Fully Convolutional Neural Network-based skill classification accuracy results along the classes defined by SURG-TLX, experience and experiment outcome (D${}_{xy}$: dissector path; S${}_{xy}$: scissors path; C${}_{xy}$: clipper path) with LOOCV validation. Blue and underscore denote the best results.

Class Labels	force${}_{x}$	force${}_{y}$	force${}_{z}$	force${}_{\mathrm{xyz}}$	D${}_{\mathrm{xy}}$	S${}_{\mathrm{xy}}$	C${}_{\mathrm{xy}}$
Experience/ Mental demands/ Situational stress	0.75	0.5	0.45	0.45	0.5	0.4	0.25
Physical demands	0.35	0.5	0.55	0.4	0.6	0.6	0.6
Temporal demands	0.55	0.65	0.85	0.6	0.55	0.45	0.55
Task complexity	0.5	0.5	0.55	0.55	0.65	0.4	0.45
Distractions	0.3	0.5	0.35	0.4	0.5	0.5	0.45
Outcome	0.45	0.55	0.55	0.35	0.55	0.55	0.5

**Table 6:** Fully Convolutional Neural Network-based skill classification accuracy results along the classes defined by SURG-TLX, experience and experimental outcomes with combined inputs (D${}_{xy}$: dissector path; S${}_{xy}$: scissors path; C${}_{xy}$: clipper path) with LOOCV validation. Blue and underscore denote the best result.

Class Labels	D${}_{\mathrm{xy}}$, S${}_{\mathrm{xy}}$, C${}_{\mathrm{xy}}$, force${}_{\mathrm{xyz}}$	D${}_{\mathrm{xy}}$, S${}_{\mathrm{xy}}$, force${}_{\mathrm{xyz}}$	D${}_{\mathrm{xy}}$, S${}_{\mathrm{xy}}$, force${}_{z}$
Experience/Mental demands/ Situational stress	0.5	0.4	0.65
Physical demands	0.25	0.35	0.35
Temporal demands	0.65	0.6	0.7
Task complexity	0.4	0.55	0.5
Distractions	0.45	0.6	0.5
Outcome	0.5	0.4	0.35

## Data Availability

Not applicable.

## References

[1] Haidegger T., Sándor J., Benyó Z. (2011). Surgery in Space: The Future of Robotic Telesurgery. Surg. Endosc..

[2] Chen J., Cheng N., Cacciamani G., Oh P., Lin-Brande M., Remulla D., Gill I.S., Hung A.J. (2018). Objective Assessment of Robotic Surgical Technical Skill: A Systemic Review (accepted manuscript). J. Urol..

[3] Nagyné Elek R., Haidegger T. (2019). Robot-Assisted Minimally Invasive Surgical Skill Assessment—Manual and Automated Platforms. Acta Polytech. Hung..

[4] Levin M., McKechnie T., Khalid S., Grantcharov T.P., Goldenberg M. (2019). Automated methods of technical skill assessment in surgery: A systematic review. J. Surg. Educ..

[5] Nagy D.A., Rudas I.J., Haidegger T. Surgical data science, an emerging field of medicine. Proceedings of the 2017 IEEE 30th Neumann Colloquium.

[6] Carswell C.M., Clarke D., Seales W.B. (2005). Assessing Mental Workload during Laparoscopic Surgery. Surg. Innov..

[7] Wadhera R.K., Parker S.H., Burkhart H.M., Greason K.L., Neal J.R., Levenick K.M., Wiegmann D.A., Sundt T.M. (2010). Is the “Sterile Cockpit” Concept Applicable to Cardiovascular Surgery Critical Intervals or Critical Events? The Impact of Protocol-Driven Communication during Cardiopulmonary Bypass. J. Thorac. Cardiovasc. Surg..

[8] Shafiei S.B., Hussein A.A., Guru K.A. (2018). Dynamic Changes of Brain Functional States during Surgical Skill Acquisition. PLoS ONE.

[9] Ahmed Y., Lone Z., Hussein A.A., Feng Y., Khan H., Broad S., Kannappan R., Skowronski A., Cole A., Wang D. (2019). Do Surgeon Non-Technical Skills Correlate with Teamwork-Related Outcomes during Robot-Assisted Surgery?. BMJ Lead..

[10] Berg R.J., Inaba K., Sullivan M., Okoye O., Siboni S., Minneti M., Teixeira P.G., Demetriades D. (2015). The Impact of Heat Stress on Operative Performance and Cognitive Function during Simulated Laparoscopic Operative Tasks. Surgery.

[11] Weigl M., Antoniadis S., Chiapponi C., Bruns C., Sevdalis N. (2015). The Impact of Intra-Operative Interruptions on Surgeons’ Perceived Workload: An Observational Study in Elective General and Orthopedic Surgery. Surg. Endosc..

[12] Lee G.I., Lee M.R., Clanton T., Clanton T., Sutton E., Park A.E., Marohn M.R. (2014). Comparative Assessment of Physical and Cognitive Ergonomics Associated with Robotic and Traditional Laparoscopic Surgeries. Surg. Endosc..

[13] Moore L.J., Wilson M.R., McGrath J.S., Waine E., Masters R.S.W., Vine S.J. (2015). Surgeons’ Display Reduced Mental Effort and Workload While Performing Robotically Assisted Surgical Tasks, When Compared to Conventional Laparoscopy. Surg. Endosc..

[14] Singh H., Modi H.N., Ranjan S., Dilley J.W.R., Airantzis D., Yang G.Z., Darzi A., Leff D.R. (2018). Robotic Surgery Improves Technical Performance and Enhances Prefrontal Activation During High Temporal Demand. Ann. Biomed. Eng..

[15] Sevdalis N., Davis R., Koutantji M., Undre S., Darzi A., Vincent C.A. (2008). Reliability of a Revised NOTECHS Scale for Use in Surgical Teams. Am. J. Surg..

[16] Hull L., Arora S., Kassab E., Kneebone R., Sevdalis N. (2011). Observational Teamwork Assessment for Surgery: Content Validation and Tool Refinement. J. Am. Coll. Surg..

[17] Yule S., Flin R., Paterson-Brown S., Maran N. (2006). Non-Technical Skills for Surgeons in the Operating Room: A Review of the Literature. Surgery.

[18] Raison N., Wood T., Brunckhorst O., Abe T., Ross T., Challacombe B., Khan M.S., Novara G., Buffi N., Van Der Poel H. (2017). Development and Validation of a Tool for Non-Technical Skills Evaluation in Robotic Surgery-the ICARS System. Surg. Endosc..

[19] Zia A., Essa I. (2018). Automated Surgical Skill Assessment in RMIS Training. Int. J. Comput. Assist. Radiol. Surg..

[20] Lajkó G., Elek R.N., Haidegger T. Surgical Skill Assessment Automation Based on Sparse Optical Flow Data. Proceedings of the IEEE International Conference on Intelligent Engineering Systems (INES).

[21] Ahn J.W., Ku Y., Kim H.C. (2019). A Novel Wearable EEG and ECG Recording System for Stress Assessment. Sensors.

[22] Koctúrová M., Juhár J. (2021). EEG-based Speech Activity Detection. Acta Polytech. Hung..

[23] Herborn K.A., Graves J.L., Jerem P., Evans N.P., Nager R., McCafferty D.J., McKeegan D.E. (2015). Skin Temperature Reveals the Intensity of Acute Stress. Physiol. Behav..

[24] Pavlidis I., Tsiamyrtzis P., Shastri D., Wesley A., Zhou Y., Lindner P., Buddharaju P., Joseph R., Mandapati A., Dunkin B. (2012). Fast by Nature—How Stress Patterns Define Human Experience and Performance in Dexterous Tasks. Sci. Rep..

[25] Di Stasi L.L., McCamy M.B., Macknik S.L., Mankin J.A., Hooft N., Catena A., Martinez-Conde S. (2014). Saccadic Eye Movement Metrics Reflect Surgical Residents’ Fatigue. Ann. Surg..

[26] Katona J. (2021). Clean and dirty code comprehension by eye-tracking based evaluation using GP3 eye tracker. Acta Polytech. Hung..

[27] Laetitia Lisetti C.L., Nasoz F. (2004). Using Noninvasive Wearable Computers to Recognize Human Emotions from Physiological Signals. EURASIP J. Appl. Signal Process..

[28] Mylonas G.P., Kwok K.W., James D.R.C., Leff D., Orihuela-Espina F., Darzi A., Yang G.Z. (2012). Gaze-Contingent Motor Channelling, Haptic Constraints and Associated Cognitive Demand for Robotic MIS. Med. Image Anal..

[29] Heemskerk J., Zandbergen H.R., Keet S.W.M., Martijnse I., van Montfort G., Peters R.J.A., Svircevic V., Bouwman R.A., Baeten C.G.M.I., Bouvy N.D. (2014). Relax, It’s Just Laparoscopy! A Prospective Randomized Trial on Heart Rate Variability of the Surgeon in Robot-Assisted versus Conventional Laparoscopic Cholecystectomy. Dig. Surg..

[30] D’Ettorre C., Mariani A., Stilli A., Rodriguez y Baena F., Valdastri P., Deguet A., Kazanzides P., Taylor R.H., Fischer G.S., DiMaio S.P. (2021). Accelerating Surgical Robotics Research: A Review of 10 Years With the Da Vinci Research Kit. IEEE Robot. Autom. Mag..

[31] Haidegger T., Speidel S., Stoyanov D., Satava R. (2022). Robot-assisted minimally invasive surgery—Surgical robotics in the data age. Proc. IEEE.

[32] Wilson M.R., Poolton J.M., Malhotra N., Ngo K., Bright E., Masters R.S.W. (2011). Development and Validation of a Surgical Workload Measure: The Surgery Task Load Index (SURG-TLX). World J. Surg..

[33] Nagyné Elek R., Haidegger T. (2021). Non-Technical Skill Assessment and Mental Load Evaluation in Robot-Assisted Minimally Invasive Surgery. Sensors.

[34] Lukezic A., Vojir T., Cehovin Zajc L., Matas J., Kristan M. Discriminative correlation filter with channel and spatial reliability. Proceedings of the IEEE Conference on Computer Vision and Pattern Recognition.

[35] Wang Z., Yan W., Oates T. Time series classification from scratch with deep neural networks: A strong baseline. Proceedings of the 2017 International Joint Conference on Neural Networks (IJCNN).

[36] Hassler K.R., Collins J.T., Philip K., Jones M.W. (2022). Laparoscopic Cholecystectomy. StatPearls.

[37] Laparoscopyboxx | Laparoscopic Training Boxes & Instruments. https://laparoscopyboxx.com/.

[38] Gao Y., Vedula S.S., Reiley C.E., Ahmidi N., Varadarajan B., Lin H.C., Tao L., Zappella L., Béjar B., Yuh D.D. (2014). Jhu-isi Gesture and Skill Assessment Working Set (jigsaws): A Surgical Activity Dataset for Human Motion Modeling. https://cirl.lcsr.jhu.edu/wp-content/uploads/2015/11/JIGSAWS.pdf.

